# Three-Dimensional Single Random Phase Encryption

**DOI:** 10.3390/s24061952

**Published:** 2024-03-19

**Authors:** Byungwoo Cho, Myungjin Cho

**Affiliations:** School of ICT, Robotics, and Mechanical Engineering, IITC, Hankyong National University, 327 Chungang-ro, Anseong 17579, Kyonggi-do, Republic of Korea; cnb7494@gmail.com

**Keywords:** integral imaging, single random phase encryption, volumetric computational reconstruction

## Abstract

In this paper, we propose a new optical encryption technique that uses the single random phase mask. In conventional optical encryptions such as double random phase encryption (DRPE), two different random phase masks are required to encrypt the primary data. For decryption, DRPE requires taking the absolute value of the decrypted data because it is complex-valued. In addition, when key information is revealed, the primary data may be reconstructed by attackers. To reduce the number of random phase masks and enhance the security level, in this paper, we propose single random phase encryption (SRPE) with additive white Gaussian noise (AWGN) and volumetric computational reconstruction (VCR) of integral imaging. In our method, even if key information is known, the primary data may not be reconstructed. To enhance the visual quality of the decrypted data by SRPE, multiple observation is utilized. To reconstruct the primary data, we use VCR of integral imaging because it can remove AWGN by average effect. Thus, since the reconstruction depth can be another key piece of information of SRPE, the security level can be enhanced. In addition, it does not require taking the absolute value of the decrypted data for decryption. To verify the validity of our method, we implement the simulation and calculate performance metrics such as peak sidelobe ratio (PSR) and structural similarity (SSIM). In increasing the number of observations, SSIM for the decrypted data can be improved dramatically. Moreover, even if the number of observations is not enough, three-dimensional (3D) data can be decrypted by SRPE at the correct reconstruction depth.

## 1. Introduction

Optical encryption, such as double random phase encryption (DRPE) [1,2,3,4,5,6,7,8,9,10,11,12,13], has been an important technique for protecting the private data recently. It can encrypt the primary data by using a 4*f* imaging system with two different random phase masks. For encryption in DRPE, the primary data are multiplied by the first random phase mask, which follows uniform distribution with range [0, 2π]. Then, it passes through the first imaging lens in the 4*f* imaging system, which means Fourier transform. Now, it is in the spatial frequency domain. After multiplying it by the second random phase mask, which also follows uniform distribution with range [0, 2π], it passes through the second imaging lens in the 4*f* imaging system, which means the inverse Fourier transform. Finally, the encrypted data can be generated. For decryption in DRPE, the encrypted data passes through the first imaging lens in the 4*f* imaging system, and it is multiplied by the complex conjugate of the second random phase mask (i.e., key information) used in the encryption of DRPE. Then, it passes through the second imaging lens in the 4*f* imaging system and is recorded by an image sensor such as a charge-coupled device (CCD). Finally, the decrypted data can be obtained. Thus, the processing speed of DRPE is the same as the speed of light. However, DRPE has two main drawbacks. First, for encryption, it needs to record the complex-valued data because it uses Fourier transform. It is difficult to obtain this complex-valued data by the conventional image sensor. To solve this problem, the holographic recording technique may be utilized since it can record both the amplitude and phase data by the conventional image sensor. Second, when attackers know the key information (i.e., the complex conjugate of the second random phase mask), the primary data may be revealed easily. To overcome this problem, photon-counting DRPE [14,15,16], three-dimensional (3D) photon-counting DRPE [17], and artificially intelligence (AI) approach [18] were proposed.

Photon-counting DRPE uses computational photon-counting imaging for recording the amplitude of the encrypted data in the encryption of DRPE. This means that encrypted data by photon-counting DRPE has more sparse amplitudes than the conventional DRPE. Thus, even though the key data are attacked, the primary data may not be recognized by human eyes. It may be recognized by matched filters such as a correlation filter, where receivers must know the primary data. Thus, it is not practical. To overcome this problem, a 3D photon-counting DRPE was proposed. It utilizes integral imaging to record the encrypted data and reconstruct the decrypted data. Since integral imaging can record multiple 2D images with different perspectives from 3D objects at a certain depth, using volumetric computational reconstruction (VCR) [19,20] and the statistical estimation methods [17] such as maximum likelihood estimation (MLE) or Bayesian approaches, the decrypted data can be reconstructed. In addition, the reconstruction depth can be another key data in 3D photon-counting DRPE. It is apparent that the primary data can be decrypted when both the phase information and the reconstruction depth are known. Therefore, it can enhance the security level of DRPE.

However, DRPE still requires two different random phase masks for encryption. In addition, for decryption, it needs taking the absolute value of the decrypted data because the decrypted data is complex-valued. To solve these problems, in this paper, we propose a new optical encryption method which uses the additive white Gaussian noise (AWGN) with zero mean and unit variance, and the single random phase mask after the first imaging lens (i.e., after Fourier transform). It can decrypt the primary data without taking the absolute value of the decrypted data, and its key information is still the same as that of DRPE. Moreover, it can enhance the security level because its 2D decrypted data may seem to be noisy data caused by AWGN. However, the 3D decrypted data can be reconstructed by VCR at the reconstruction depth since VCR has the average effect. This means that the 2D decrypted data are overlapping each other so that the average value of the overlapped noise data goes to zero by AWGN. To verify the validity of our proposed method, we implement the simulation and calculate peak sidelobe ratio (PSR) and structural similarity (SSIM) as the performance metric.

This paper consists of the following sections. We present the basic concept of DRPE and VCR integral imaging in Section 2. Then, we describe our proposed method, which is called single random phase encryption (SRPE) in Section 3. Simulation results are shown in Section 4. Finally, we conclude with future work in Section 5.

## 2. Three-Dimensional Double Random Phase Encryption

### 2.1. Double Random Phase Encryption

**Figure 1:**
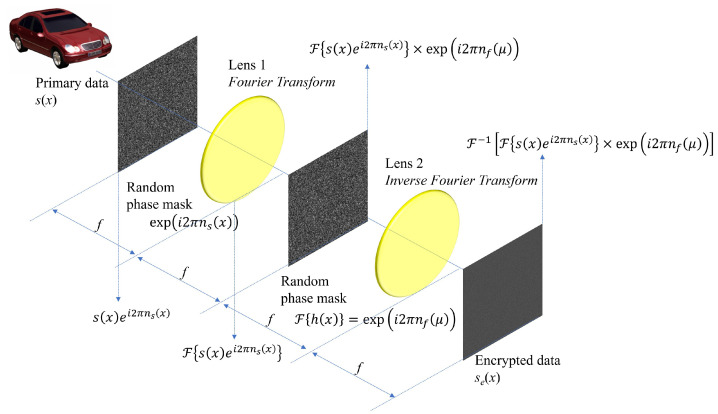
Encryption of double random phase encryption.

Double random phase encryption (DRPE), which is one of optical encryption methods, can encrypt primary data by using an optical imaging system such as the 4*f* imaging system. Its encryption and decryption speed is the same as the speed of light. Figure 1 illustrates the encryption process of DRPE. First, the primary data s(x) pass through the first random phase mask defined as $e^{i2\pi n_{s}\left(x\right)}$, where the phase in spatial domain $n_{s}\left(x\right)$ follows uniform distribution with range [0, 1]. Then, $s\left(x\right)e^{i2\pi n_{s}\left(x\right)}$ is generated. It enters the first imaging lens, and its domain is converted into the spatial frequency domain as $F\{s\left(x\right)e^{i2\pi n_{s}\left(x\right)}\}$, which means Fourier transform. At the focal plane after the first imaging lens, it can be multiplied by the second random phase mask defined as $F\left\{h\left(x\right)\right\}=e^{i2\pi n_{f}\left(\mu \right)}$. Thus, $F\left\{s\left(x\right)e^{i2\pi n_{s}\left(x\right)}\right\}\times e^{i2\pi n_{f}\left(\mu \right)}$ can be obtained. Finally, it passes through the second imaging lens, which means the inverse Fourier transform and the encrypted data $s_{e}\left(x\right)$ can be acquired. The encryption process can be written as the following [17]

(1) $$s_{e}\left(x\right)=F^{-1}\left[F\left\{s\left(x\right)e^{i2\pi n_{s}\left(x\right)}\right\}\times e^{i2\pi n_{f}\left(\mu \right)}\right]$$

where F and $F^{-1}$ are Fourier transform and inverse Fourier transform, respectively.

**Figure 2:**
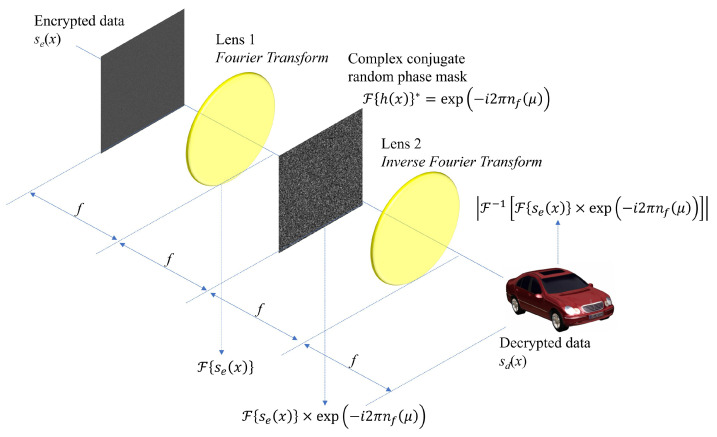
Decryption of double random phase encryption.

**Figure 3:**
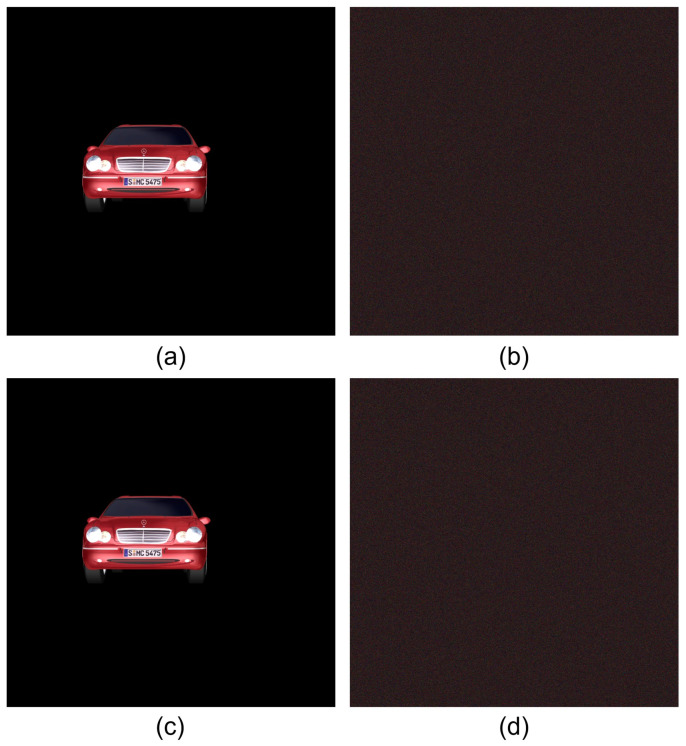
Double random phase encryption results. (**a**) Primary data, (**b**) encrypted data, (**c**) decrypted data with correct key information, and (**d**) decrypted data with incorrect key information.

The encrypted data $s_{e}\left(x\right)$ are complex-valued, and the conjugate of the second random phase mask $F{\left\{h\left(x\right)\right\}}^{*}=e^{-i2\pi n_{f}\left(\mu \right)}$ is the key information in DRPE. Using them, the primary data can be decrypted. Figure 2 presents the decryption process of DRPE with the key information. In the decryption process, a 4*f* imaging system is still utilized. The encrypted data $s_{e}\left(x\right)$ pass through the first imaging lens, and its domain is converted into the spatial frequency domain as $F\{s_{e}\left(x\right)\}$. Then, it is multiplied by the key information and passes through the second imaging lens (i.e., the inverse Fourier transform). Finally, the decrypted data $s_{d}\left(x\right)$ can be obtained by an image sensor such as the charge-coupled device (CCD). The following equation describes the decryption process [17].

(2) $$s_{d}\left(x\right)=\left|F^{-1}\left[F\left\{s_{e}\left(x\right)\right\}\times e^{-i2\pi n_{f}\left(\mu \right)}\right]\right|$$

where |·| is the absolute value operator. In DRPE, the key information should be protected from attackers because the primary data can be decrypted easily when the key information is revealed. Figure 3 shows results of DRPE. The primary data as shown in Figure 3a can be encrypted by Equation (1). As shown in Figure 3b, the encrypted data seem to be noisy data and cannot be recognized. Using Equation (2) with the correct key information, the decrypted data as shown in Figure 3c can be obtained. In fact, it is the same as the primary data as shown in Figure 3a. However, when the incorrect key information is used, the decrypted data as shown in Figure 3d cannot be recognized. Therefore, the key information is the most important factor in DRPE. To improve the security level in DRPE, integral imaging, which is one of 3D imaging techniques, can be applied to DRPE. In next subsection, 3D DRPE is presented.

### 2.2. Integral Imaging and Three-Dimensional Double Random Phase Encryption

**Figure 4:**
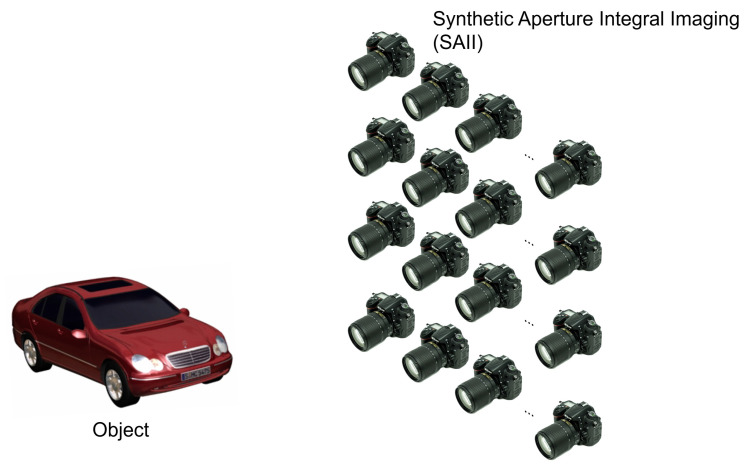
Synthetic aperture integral imaging.

Integral imaging is a passive 3D imaging technique, which can capture multiple 2D images with different perspectives through lens array or camera array and obtains 3D images by optical display or computational reconstruction such as volumetric computational reconstruction (VCR). Multiple 2D images with different perspectives in integral imaging are referred to as elemental images. In lens array based integral imaging, each elemental image has a few pixels since the number of pixels in the image sensor is divided by the number of lenses in lens array. It may cause the degradation of the resolution for 3D images. To overcome this problem, synthetic aperture integral imaging (SAII) [21] was proposed. Figure 4 illustrates SAII, which uses camera array. In SAII, each elemental image has the same number of pixels as the image sensor. Hence, 3D image with high quality can be obtained. In this paper, we use SAII to record elemental images.

**Figure 5:**
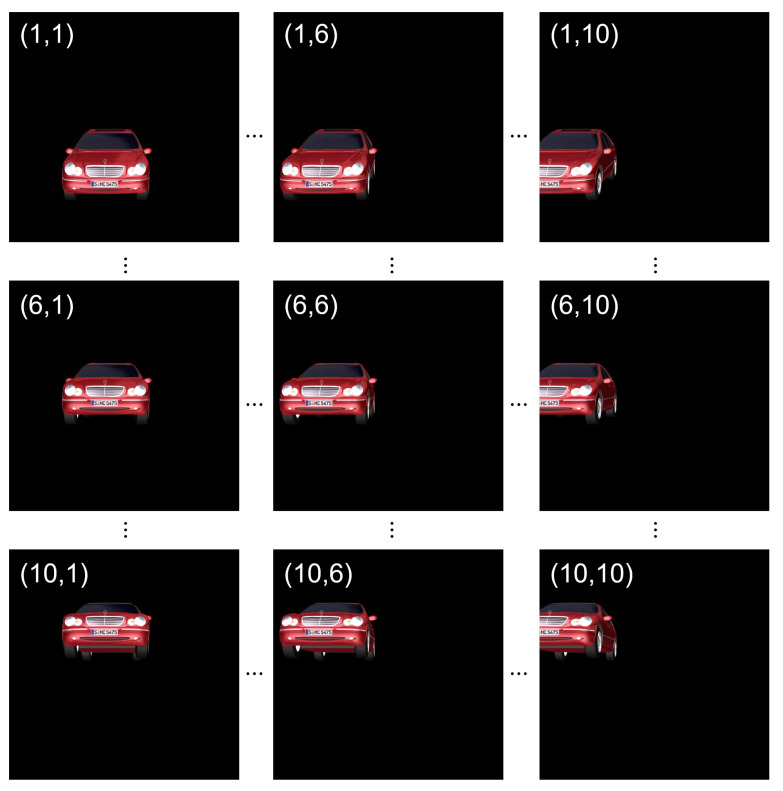
Elemental images captured by SAII.

Figure 5 shows several elemental images. In this paper, we captured 10 (H) × 10 (V) elemental images with 1000 (H) × 1000 (V) pixels. The 3D object is a car located 323 mm from the camera array. In Figure 5, (row, column) means index of each elemental image. As shown in Figure 5, each elemental image has different perspective.

**Figure 6:**
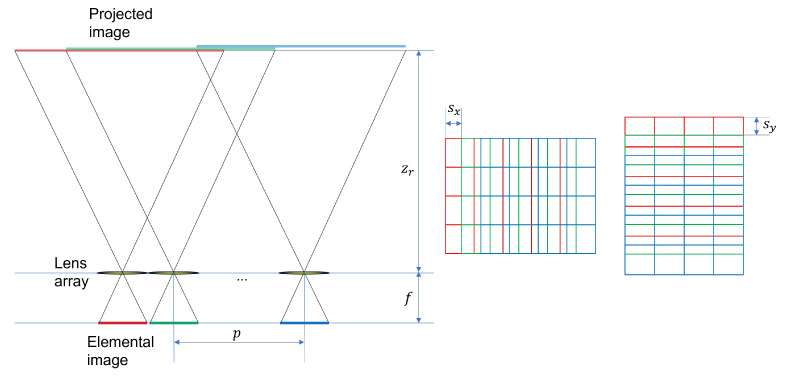
Volumetric computational reconstruction.

Now, a 3D image can be reconstructed by the VCR. Figure 6 illustrates the concept of VCR. First, each elemental image is back-projected through the lens array, where the distance between the elemental images and the lens array is the same as the focal length of the camera array (*f*) used in SAII. The pitch between elemental images is *p*, and the reconstruction depth is $z_{r}$. On the reconstruction plane, the projected images are overlapping each other. Thus, it is required to calculate the shifting pixels between the projected images such as $s_{x}$ and $s_{y}$. The following equations present the process of VCR [19,20].

(3) $$\begin{array}{c} \begin{array}{cc} s_{x}\left(k\right) & =\left\lceil \frac{N_{x}fp_{x}}{c_{x}z_{r}}\times \left(k-1\right)\right\rfloor ,\;k=1,2,\ldots ,K\\ s_{y}\left(l\right) & =\left\lceil \frac{N_{y}fp_{y}}{c_{y}z_{r}}\times \left(l-1\right)\right\rfloor ,\;l=1,2,\ldots ,L \end{array} \end{array}$$

(4) $$I\left(x,y,z_{r}\right)=\frac{1}{O(x,y,z_{r})}\sum_{k=1}^{K}\sum_{l=1}^{L}I_{kl}\left(x+s_{x}\left(k\right),y+s_{y}\left(l\right)\right)$$

where $N_{x},N_{y}$ are the number of pixels for each elemental image in *x* and *y* directions, *f* is the focal length of the camera, $p_{x},p_{y}$ are the pitch between elemental images in *x* and *y* directions, $c_{x},c_{y}$ are the size of the image sensor in *x* and *y* directions, $z_{r}$ is the reconstruction depth, k,l is the indices of each elemental images in *x* and *y* directions, K,L are the total number of elemental images in *x* and *y* directions, $I_{kl}$ is the *k*th column and *l*th row elemental image, $O(x,y,z_{r})$ is the overlapping matrix at the reconstruction depth $z_{r}$, and $I(x,y,z_{r})$ is the reconstructed 3D image at the reconstruction depth $z_{r}$, respectively. Here, the shifting pixels are approximated by rounding operator ⌈·⌋ because the shifting pixels are integer-valued.

**Figure 7:**
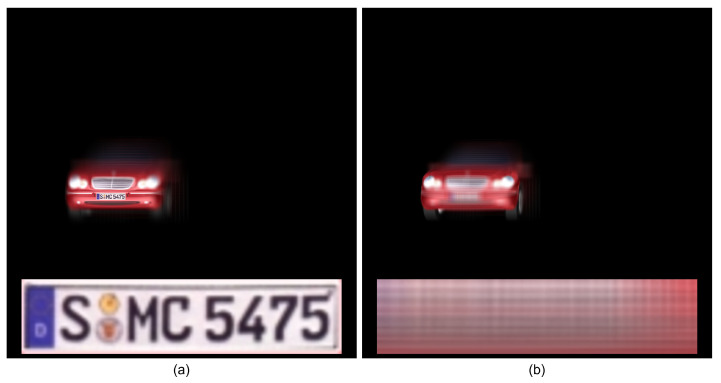
Results by 3D DRPE at (**a**) 323 mm and (**b**) 350 mm.

Using Equations (3) and (4) with elemental images as shown in Figure 5, the reconstructed 3D images can be obtained. Now, integral imaging and DRPE can be merged to enhance the security level, where it is called 3D DRPE. First, using Equations (1) and (2) with elemental images as shown in Figure 5, the encrypted and decrypted data can be obtained. Then, using Equations (3) and (4) with the decrypted data, 3D images can be reconstructed as shown in Figure 7. Since the license plate of the car is located at 323 mm, Figure 7a shows better image quality than Figure 7b. This means that the reconstruction depth can be another key information in 3D DRPE so that it can improve the security level of DRPE. However, it still has drawbacks. If attackers know both the key information and the reconstruction depth, they can observed the primary data. In addition, it requires two different random phase masks for encryption. To solve these problems, in this paper, we propose the single random phase encryption (SRPE), which uses the single random phase mask and AWGN. In the next section, we present our proposed method in detail.

## 3. Three-Dimensional Single Random Phase Encryption

In this section, we describe our proposed method step by step. First, we explain the encryption process of SRPE. Then, we present how to decrypt the encrypted data by SRPE. Finally, to improve the security level of SRPE, we suggest 3D SRPE.

### 3.1. Principle of Three-Dimensional Single Random Phase Encryption

**Figure 8:**
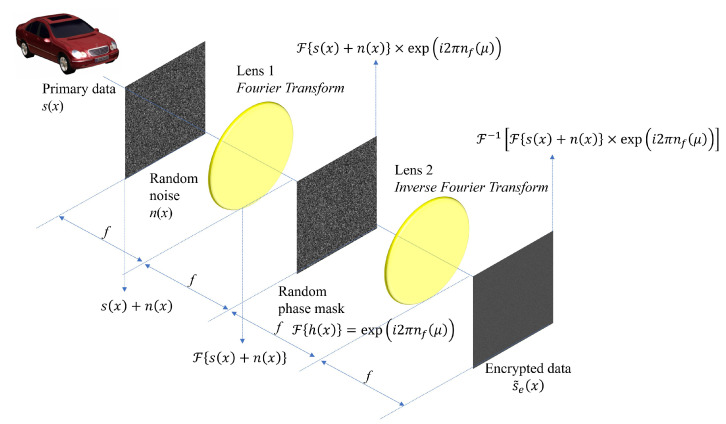
Encryption of single random phase encryption.

Figure 8 illustrates the encryption process of SRPE. It uses the same 4*f* imaging system as DRPE. In our proposed method, the random noise in spatial domain n(x) is introduced. This random noise is additive white Gaussian noise (AWGN), that is, it follows Normal distribution with zero mean and unit variance N(0,1). Thus, the primary data with AWGN s(x)+n(x) can be obtained. Then, it passes through the first imaging lens, and its domain is converted into the spatial frequency domain as F{s(x)+n(x)}. In SRPE, the same key information as DRPE $F\left\{h\left(x\right)\right\}=e^{i2\pi n_{f}\left(\mu \right)}$, is used. Finally, by the same manner as DRPE, the encrypted data ${\tilde{s}}_{e}\left(x\right)$ can be obtained as the following:

(5) $${\tilde{s}}_{e}\left(x\right)=F^{-1}\left[F\left\{s(x)+n(x)\right\}\times e^{i2\pi n_{f}\left(\mu \right)}\right]$$

Here, the single random phase mask is used in SRPE. Thus, its encryption process is simpler than DRPE. In addition, even if attackers know the key information, the decrypted data may not be recognized because of AWGN.

**Figure 9:**
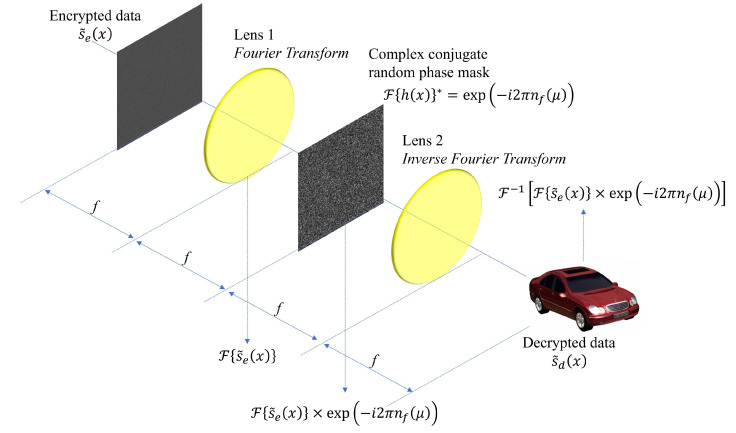
Decryption of single random phase encryption.

Now, the encrypted data are decrypted through the decryption of SRPE as illustrated in Figure 9. The encrypted data ${\tilde{s}}_{e}\left(x\right)$ passes through the same 4*f* imaging system as the decryption process of DRPE. Here, the key information $F{\left\{h\left(x\right)\right\}}^{*}=e^{-i2\pi n_{f}\left(\mu \right)}$ is also the same as DRPE. Finally, the decrypted data can be obtained by the following

(6) $${\tilde{s}}_{d}\left(x\right)=F^{-1}\left[F\left\{{\tilde{s}}_{e}\left(x\right)\right\}\times e^{-i2\pi n_{f}\left(\mu \right)}\right]$$

Here, the absolute value operator |·| is not used because SRPE does not use the first random phase mask in DRPE. It is another advantage of SRPE compared with DRPE.

**Figure 10:**
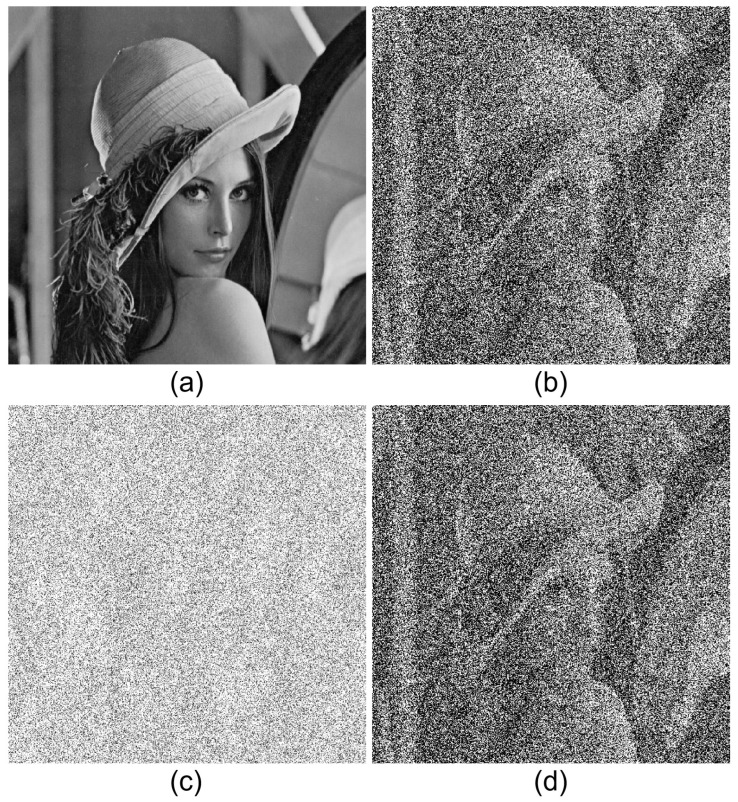
Results by single random phase encryption. (**a**) Primary data, (**b**) primary data with AWGN, (**c**) encrypted data, and (**d**) decrypted data with correct key information.

Figure 10 shows the results by SRPE, where the lena test image is used as the primary data. Figure 10b is the primary data with AWGN, and Figure 10c is the encrypted data by Equation (5). Figure 10d is the decrypted data with the correct key information. It is noticed that Figure 10b,d is the same, and they seem to be noisy data. This means that even if attackers know the key information, the decrypted data cannot be recognized.

**Figure 11:**
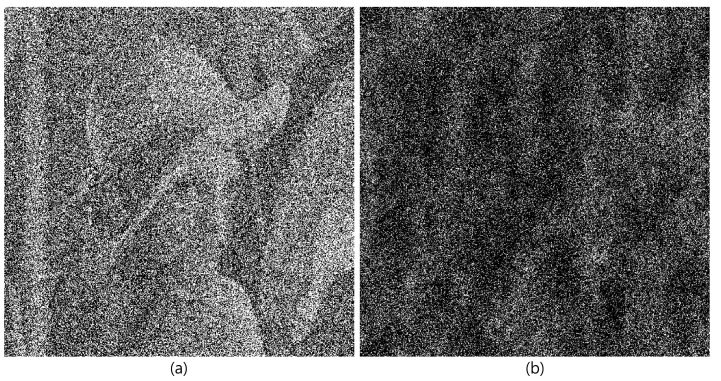
Decrypted data with (**a**) correct key information and (**b**) incorrect key information.

Figure 11 shows the comparison for decrypted data with correct and incorrect key information. As shown in Figure 11b, when the key information is not correct, the decrypted data cannot be recognized.

To improve the visual quality of the decrypted data with correct key information in SRPE, the characteristics of AWGN can be utilized. Since AWGN follows normal distribution with zero mean and unit variance N(0,1), the visual quality of the decrypted data can be enhanced by generating decrypted data from multiple encrypted data and taking the expectation of multiple decrypted data. This process can be written as the following

(7) $${\tilde{s}}_{\bar{d}}\left(x\right)=E\left[{\tilde{s}}_{d_{n}}\left(x\right)\right],\;n=1,2,3,\ldots ,N$$

where ${\tilde{s}}_{d_{n}}\left(x\right)$ is the *n*th decrypted data generated by the SRPE decryption process, E[·] is the expectation operator, *N* is the total number of generation of the decrypted data, and ${\tilde{s}}_{\bar{d}}\left(x\right)$ is the averaged decrypted data with improved visual quality.

**Figure 12:**
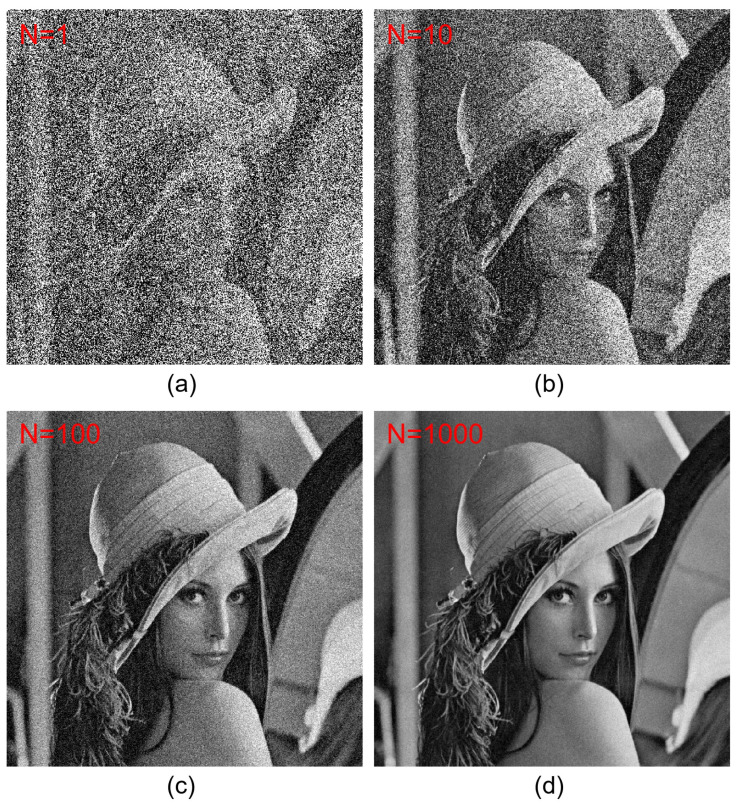
Decrypted data with improved visual quality by expectation and multiple decrypted data in SRPE. (**a**) N=1, (**b**) N=10, (**c**) N=100, and (**d**) N=1000, where *N* is the number of SRPE generation.

Figure 12 shows the decrypted data by expectation and multiple decrypted data in SRPE. As increasing the number of generations for SRPE, the visual quality of the decrypted data can be improved since AWGN has zero mean. In addition, as mentioned in the 3D DRPE, integral imaging can be utilized in SRPE because the reconstruction depth can be another key piece of information. Therefore, 3D SRPE can be written as follows:

(8) $$\tilde{I}\left(x,y,z_{r}\right)=\frac{1}{O(x,y,z_{r})}\sum_{k=1}^{K}\sum_{l=1}^{L}{\tilde{s}}_{\bar{d}kl}\left(x+s_{x}\left(k\right),y+s_{y}\left(l\right)\right)$$

where ${\tilde{s}}_{\bar{d}kl}$ is the mean of multiple *k*th column and *l*th row decrypted elemental images. To verify the validity of our proposed method, we show the simulation results in the next section.

### 3.2. Computational Procedure of Three-Dimensional Single Random Phase Encryption

To explain the computational implementation of SRPE in more detail, the following procedures are shown in (Algorithm 1).
**Algorithm 1:** Single Random Phase Encryption**procedure** (enc, key) := SRPE_enc (in_data : input data, ratio : noise ratio) **defined as:**    n := ratio × **randn**(**size**(in_data))    mask := exp(i2π× **rand**(**size**(in_data)))    enc := **ifft**(**fft**(in_data + n)×mask)    key := **conj**(mask)**end procedure****procedure** dec := SRPE_dec (enc : encrypted data, key : key information) **defined as:**    dec := **ifft**(**fft**(enc)×key)**end procedure**
where **randn** and **rand** are the random generation from normal distribution and uniform distribution, respectively. In addition, **fft**, **ifft**, and **conj** are Fourier transform, inverse Fourier transform, and complex conjugate, respectively. Therefore, using these two procedures, SRPE can be implemented computationally.

## 4. Simulation Results

To show the ability of 3D SRPE, we recorded multiple images with different perspectives (i.e., elemental images) by SAII. A 10 (H) × 10 (V) camera array is used, where the focal length *f* is 50 mm, the pitch between elemental images *p* is 10 mm in both *x* and *y* directions, the sensor size is 36 (H) mm × 36 (V) mm, and the resolution of each elemental image is 1000 (H) × 1000 (V) pixels. The 3D object is a car with the licence plate “SMC 5475” which is located 323 mm from the camera array. AWGN follows normal distribution with zero mean and unit variance N(0,1). We generated 1000 encrypted data randomly by Equation (5) for each elemental image. Then, the decrypted data were obtained by Equation (6). To improve the visual quality of the decrypted data, we used Equation (7) with 1000 decrypted data for each elemental image. Finally, using Equations (3) and (8) with 10 (H) × 10 (V) elemental images, the reconstructed 3D images at 323 mm was obtained.

**Figure 13:**
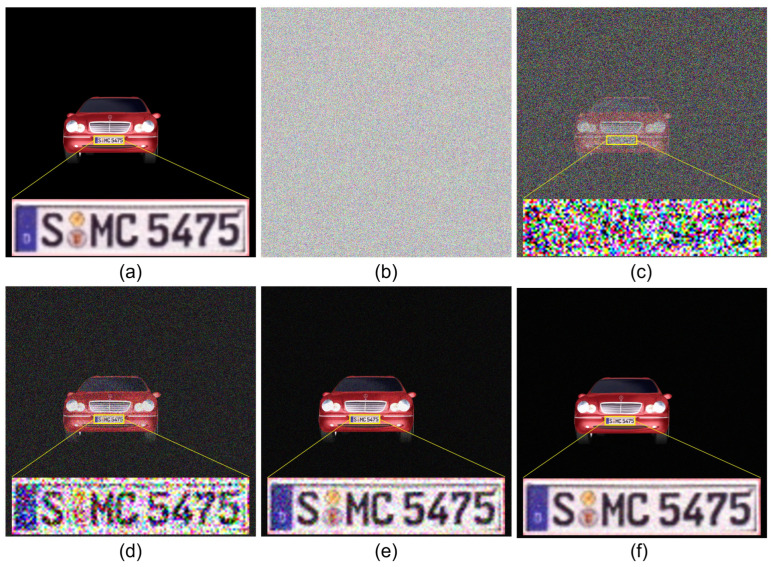
2D results by SRPE. (**a**) Primary data, (**b**) encrypted data, (**c**) decrypted data with N=1, (**d**) decrypted data with N=10, (**e**) decrypted data with N=100, and (**f**) decrypted data with N=1000, where *N* is the number of generation for SRPE.

Figure 13 shows 2D results by SRPE. To compare the visual quality of the decrypted data, the license plate is enlarged for each decrypted result. Figure 13a is the primary data, which are used as the reference. Figure 13b is the encrypted data by SRPE. Figure 13c–f shows the decryption results via various generations (i.e., N=1, N=10, N=100, and N=1000) of SRPE. As shown in Figure 13, it is apparent that the visual quality of the decrypted data depends on the number of generations of SRPE. Thus, the result shown in Figure 13f has the best visual quality compared to the others. For numerical comparison, we calculated the structural similarity (SSIM) as shown in Table 1. It is noticed that the similarity can be improved by increasing the number of generations of SRPE. However, when the number of generations of SRPE increases, the processing speed of SRPE is slow. Therefore, to obtain the reasonable visual quality and processing speed, 3D SRPE was implemented.

**Figure 14:**
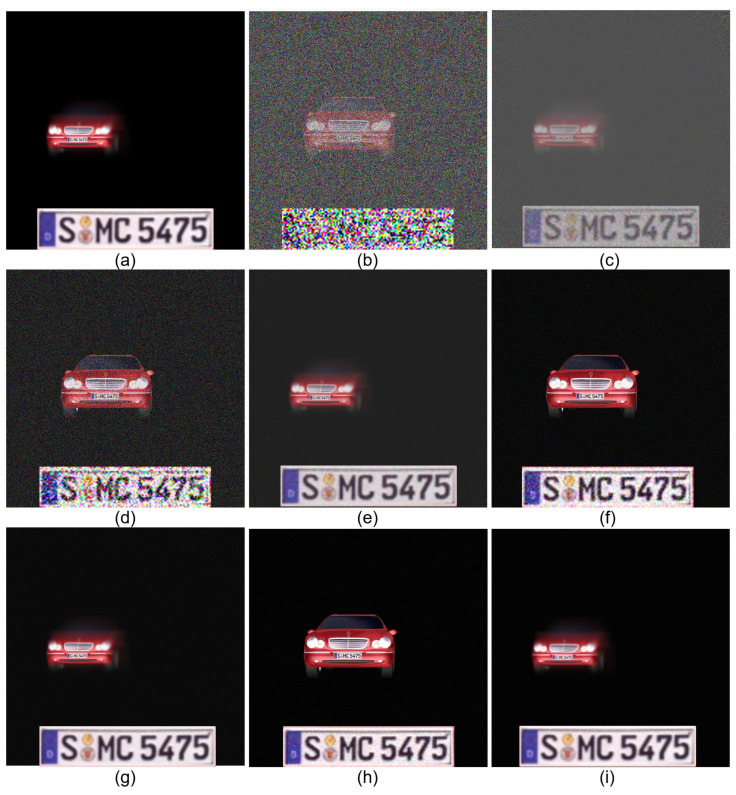
Comparison between 2D and 3D results by SRPE. (**a**) 3D primary data, (**b**) 2D decrypted data with N=1, (**c**) 3D decrypted data with N=1, (**d**) 2D decrypted data with N=10, (**e**) 3D decrypted data with N=10, (**f**) 2D decrypted data with N=100, (**g**) 3D decrypted data with N=100, (**h**) 2D decrypted data with N=1000, and (**i**) 3D decrypted data with N=1000.

Figure 14 shows 2D and 3D results by SRPE via various generations of SRPE. It is noticed that 3D results have better visual quality than 2D results. In addition, as we increase the number of generations of SRPE, the visual quality of the decrypted data is improved. However, even though the number of generations of SRPE increases, the visual quality of the decrypted data is limited (i.e., saturation). For numerical comparison, we calculated the SSIM and SSIM ratio between the 2D and 3D results as shown in Table 2. When N=1, the 3D results have 4.68 times the SSIM than the 2D results. It is remarkable that the visual quality of 3D results with N=1 is dramatically improved compared to the others. This means that a lot of generations of SRPE are not required. In addition, when N=1000, SSIM is 1, which means that the primary data and the decrypted data are the same as each other (i.e., perfect decryption).

In 3D SRPE, since the reconstruction depth is another key information, we need to show that the primary data are revealed at the only correct reconstruction depth. Thus, we found the correlation between the primary data and the decrypted data via different reconstruction depths by using *k*th low nonlinear correlation filter [22]. It has non-linearity factor *k*, which is 0<k<1 real number. The filter is defined as the following [22]:

(9) $$c\left(x,y\right)={\left|F^{-1}\left[{\left(|R(x,y)||T(x,y)|\right)}^{k}\exp\left\{i\left(\phi_{T}-\phi_{R}\right)\right\}\right]\right|}^{2}$$

where |R(x,y)|,|T(x,y)| are the amplitudes of Fourier transformed reference and target images, $\phi_{R},\phi_{T}$ are the phases of Fourier transformed reference and target images, and c(x,y) is the correlation result between the reference and target images, respectively.

In addition, for numerical analysis, peak sidelobe ratio (PSR) was calculated by the following [22]

(10) $$PSR=\frac{c_{max}-\bar{c}}{\sigma_{c}}$$

where $c_{max}$ is the maximum value of the correlation result by Equation (9), $\bar{c}$ is the mean value of the correlation result, and $\sigma_{c}$ is the standard deviation of the correlation result. When PSR value is high, the correlation is strong.

**Figure 15:**
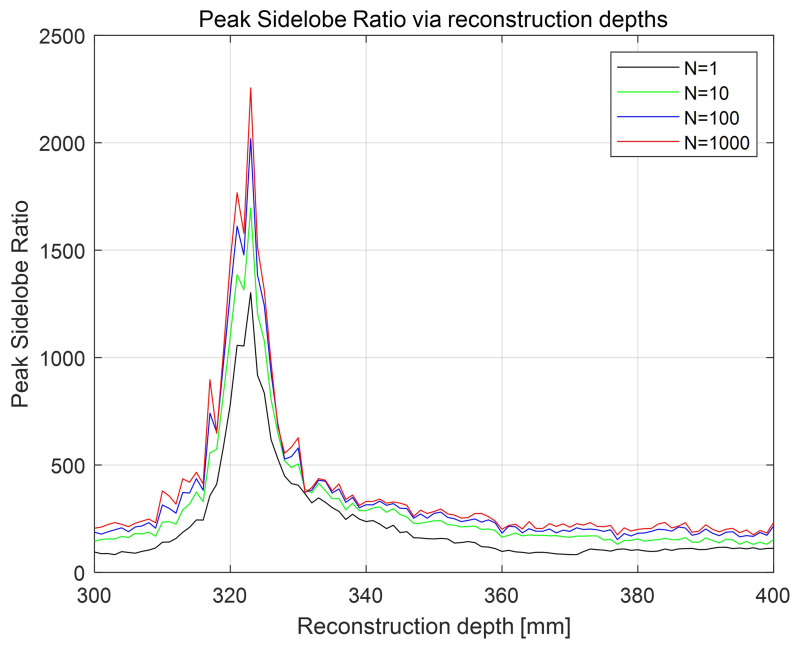
Peak sidelobe ratio for the decrypted data with various generations of SRPE via different reconstruction depths.

Figure 15 shows PSR results for the decrypted data with various generations of SRPE via different reconstruction depths. Here, the reference image is the 3D reconstructed image at 323 mm obtained by using VCR and elemental images, as shown in Figure 5. All decrypted data have the highest PSR at 323 mm. When N=1, PSR value at 323 mm is 1302.358209. On the other hand, when N=1000, PSR value at 323 mm is 2255.662650. This means that the reconstruction depth can be another key information in SRPE.

Moreover, for the encryption efficiency comparison, we measured the processing time between DRPE and SRPE. System specification used for comparison is shown in Table 3. The 10 (H) × 10 (V) elemental images, as shown in Figure 5, which has 1000 (H) × 1000 (V) color pixels, are encrypted and decrypted. For SRPE, the number of observations is set as 1 and 10. The processing time is shown in Table 4.

As shown in Table 4, for single observation, the encryption and decryption processing time of SRPE is slightly less than that of DRPE. However, since SRPE requires multiple observations to improve the visual quality of the decrypted data, the processing time of SRPE is much more than DRPE for multiple observations. It is limitation of SRPE.

## 5. Conclusions

In this paper, we have proposed a new optical 3D encryption method which uses the single random phase mask and AWGN. Our method, SRPE, can encrypt the primary data through the same 4*f* imaging system as DRPE by using the single random phase mask and AWGN. The decryption process of SRPE is almost the same as DRPE, but it does not need the absolute value operator. When attackers know the key information, the primary data can be revealed easily in DRPE. On the other hand, in SRPE, it is difficult to observe the primary data because the decrypted data still have noise caused by AWGN. To improve the visual quality of the decrypted data in SRPE, expectation operator can be utilized. In addition, to enhance the security level in SRPE, integral imaging can be applied. Thus, the reconstruction depth can be another key information. In our proposed method, the primary data can be revealed when attackers knows the key information, the reconstruction depth, and multiple decrypted data. Thus, it may be impossible to observe the primary data in SRPE. Therefore, we believe that our method can be used for various applications that consider the private information. However, our method has several drawbacks. To encrypt the primary data, SRPE needs a lot of encrypted data for decryption. Thus, its processing speed is a critical problem. In addition, it needs a method to record the complex-valued data because the encrypted data are complex-valued. In our opinion, this may be solved by introducing the holography technique. We will investigate these issues in future work. 

## Figures and Tables

**Table 1:** Structural similarity (SSIM) for 2D decryption results via various generations of SRPE.

N=1	N=10	N=100	N=1000
0.1319	0.4893	0.8710	0.9823

**Table 2:** Structural similarity (SSIM) comparison between 2D and 3D results via various generations of SRPE.

	2D	3D	3D/2D (Times)
*N* = 1	0.1319	0.6176	4.68
*N* = 10	0.4893	0.9536	1.95
*N* = 100	0.8710	0.9968	1.14
*N* = 1000	0.9823	1.0000	1.02

**Table 3:** System specification used for the processing time comparison.

CPU	RAM	Software	OS
AMD Ryzen 7 3700X	32 GB	MATLAB 2023a	Windows 11 Pro

**Table 4:** Encryption and decryption processing time between DRPE and SRPE.

Generation	Elemental Images	DRPE		SRPE	
		Encryption (Second)	Decryption (Second)	Encryption (Second)	Decryption (Second)
*N* = 1	1	0.217534	0.0863	0.211983	0.0773
	100	17.666209	8.0791	16.533440	7.5473
*N* = 10	1			1.589549	0.7458
	100			153.771801	73.8643

## Data Availability

All data are contained within the article.

## Abbreviations

The following abbreviations are used in this manuscript:

AI	Artificially intelligence
AWGN	Additive white Gaussian noise
CCD	Charge-coupled device
DRPE	Double random phase encryption
MLE	Maximum likelihood estimation
PSR	Peak sidelobe ratio
SAII	Synthetic aperture integral imaging
SRPE	Single random phase encryption
SSIM	Structural similarity
VCR	Volumetric computational reconstruction
